# One year of general practice during the COVID-19 pandemic – presentation and evaluation of digital medical education

**DOI:** 10.3205/zma001550

**Published:** 2022-07-15

**Authors:** Piet van der Keylen, Nikoletta Zeschick, Anna-Lena Langer, Thomas Kühlein, Marco Roos

**Affiliations:** 1 Friedrich-Alexander-University Erlangen-Nürnberg, Institute of General Practice, University Hospital Erlangen, Erlangen, Germany; 2 Universität Augsburg, Medizinische Fakultät, Lehrstuhl für Allgemeinmedizin, Augsburg, Germany

**Keywords:** digital teaching, COVID-19, general practice, inverted classroom, blended learning

## Abstract

**Background and teaching situation:**

The SARS-CoV-2 pandemic had a substantial didactic impact on medical teaching. In Erlangen, the lecture “General Practice” was offered asynchronously and digitally in an inverted-classroom concept. Contents were available via a learning platform. The lecture was presented using annotated videos, consolidation materials and control questions. A forum encouraged for discussions and feedback and collected in-depth aspects for a case-based video consultation. The aim of this work is to evaluate and critically examine the digital teaching concept during the SARS-CoV-2 pandemic.

**Methodology:**

Two semester cohorts evaluated the lecture. Overall impression of the lecture, didactic elements, suitability and the desired future lecture format were surveyed quantitatively. Free text answers were evaluated by means of qualitative content synthesis.

**Results:**

In terms of overall impression, the students (*N*=199) rated the lecture on average as *“very good” *(*M*=1.41, *SD*=.57). Digital methods were perceived as suitable for supporting self-study, and digital usage was rated as unproblematically (*M*=1.18, *SD*=.50). Desired future teaching formats were blended learning concepts (79.4%). Organisation, structure and content presentation were highly appreciated. The time for completing the course was perceived critically. The students urged for more practical and consolidating lecture work.

**Discussion and implications:**

The results illustrate high acceptance of digital teaching and underline the demand for future blended learning concepts. It is particularly important to better consider the students’ time investment and practical relevance of digital self-learning mechanisms.

## Zusammenfassung

**Hintergrund und Lehrsituation:**

Die SARS-CoV-2-Pandemie wirkte sich didaktisch stark auf den medizinischen Lehrbetrieb aus. In Erlangen wurde die Vorlesung Allgemeinmedizin asynchron und digital im Inverted Classroom-Konzept angeboten. Inhalte waren über eine Lernplattform verfügbar. Die Vermittlung erfolgte mit kommentierten Videos, vertiefenden Materialien und Kontrollfragen. Ein Forum ermunterte zu Diskussion und Feedback und sammelte Vertiefungsaspekte für eine fallbasierte Videosprechstunde. Ziel der Arbeit ist die Evaluation und kritische Auseinandersetzung mit dem digitalen Lehrkonzept während der SARS-CoV-2-Pandemie.

**Methodik:**

Zwei Semesterkohorten evaluierten die Veranstaltung. Es wurden der Gesamteindruck der Veranstaltung, didaktische Elemente, deren Eignung und die künftig gewünschte Vorlesungsform quantitativ erfragt. Freitextantworten wurden mittels qualitativer Inhaltssynthese ausgewertet.

**Ergebnisse:**

Im Gesamteindruck bewerteten die Studierenden (*N*=199) die Vorlesung durchschnittlich als *„sehr gut“* (*M*=1.41, *SD*=.57). Digitale Methoden wurden als geeignet für die Unterstützung des Selbststudiums empfunden und die digitale Anwendung als problemlos bewertet (*M*=1.18, *SD*=.50). Gewünschte künftige Lehrformate waren Blended Learning-Konzepte (79.4%). Gelobt wurden Organisation, Struktur und inhaltliche Darstellung. Kritisch gesehen wurde der Zeitfaktor zum Absolvieren der Veranstaltung. Die Studierenden forderten mehr praxisnahes und vertiefendes Arbeiten.

**Diskussion und Implikationen:**

Die Ergebnisse verdeutlichten die hohe Akzeptanz digitaler Lehre und unterstreichen die Forderung nach zukünftigen Blended Learning-Konzepten. Besonders gilt es, den Zeitaufwand und Praxisnähe digitaler Selbstlernmechanismen seitens der Studierenden besser zu berücksichtigen.

## 1. Background

### 1.1. Impacts of the pandemic

The SARS-CoV-2 pandemic generated circumstances in Germany [1] that may offer potential for sustainable changes in medical teaching and didactics. One challenge was the sustainable transformation of established courses into a modern, digital format in a short time. Although the *Masterplan for Medical Studies 2020* and the German Council of Science and Humanities have long been pointing out the urgency to digitise medical teaching [2], [3], it was the SARS-CoV-2 pandemic that created the necessary pressure to take action. Medical faculties are also aware of the importance of the digitalisation of the healthcare system and the impact on the medical curriculum [4].

#### 1.2. Teaching situation

Erlangen anchors general practice in the 1^st^ clinical semester as a lecture with two semester hours per week (*Semesterwochenstunden*; SWS). Core contents are primary (general) medicine in acute low-prevalence consultations, the care of patients with common (chronic) diseases and methods of (also digital) information retrieval by the means of evidence-based medicine. Pre-pandemically, general practice was organised as a classroom lecture to convey content on the basis of consultation cases. The content had already been made digitally available beforehand via an ILIAS learning platform. Using this learning platform and its digital possibilities, the “lecture general practice” and the clinical elective “Smart decisions in everyday clinical practice” were offered fully digitally for the first time in the summer term 2020 [5], [6].The main didactic idea was to promote the digitalisation of general practice teaching in the curriculum as well [7]. Furthermore, other subjects within the medical curriculum are also showing efforts to transform digital teaching through inverted classroom models [8], [9], [10]. In addition to changing the teaching method, the complexity of the learning goals was transformed from a rather content-oriented level to a more reflective and consolidating level. With this, the heterogeneity of medical procedures in general practice is facilitated beyond factual knowledge and medical guideline recommendations. This work presents an asynchronous, fully digital concept for curricular teaching of general practice. Primary research questions of this work are:

- How do students evaluate the concept presented?
- What is the potential for improvement and what digital format do students request for the future?

Thus, approaches for further development after SARS-CoV-2 shall be compiled.

## 2. Concept and methods

The lecture content was offered asynchronously, i.e. communication and interaction between teachers and learners took place at different times. On the basis of a pilot work [6], didactic and content-related adaptations (esp. improvement of the video material, purely audio-commented presentations were now additionally video-commented and provided with further content, such as links and QR codes) were made for the subsequent terms presented here (winter term 2020/2021 and summer term 2021). 

### 2.1. Digital transformation

The time slots of the formerly 90-minute lectures were offered as video-commented recordings. For this purpose, each lecture was divided into three to five smaller, shorter topic blocks (10 to 30 minutes, *“segmenting principle”*) [11] and made available by audio or video annotated presentation (.mp4) via the ILIAS learning platform [https://www.studon.fau.de]. Total duration of the video content should not exceed 45-60 minutes per lecture to reduce content emphasis and, in the sense of the inverted classroom concept, create more time to the critical examination of content and higher learning objective complexities. From the second online semester onwards, the contents of the entire semester were already available at the beginning of the semester to enable even more individual learning independent from time and location. In order to consolidate individual topic blocks, activating assignments for self-study were integrated into the learning platform. Thereby, digital passivity was to be antagonised already during the content presentation stage. The theoretical content was now applied to patient cases and reflective discussions of guideline recommendations and research papers were introduced. After this type of content presentation, digital exercise questions were provided via the learning platform for self-monitoring, which included feedback and explanation functions. To counteract possible content-related ambiguities during self-study, an interactive forum was offered via the learning platform, where discussion for the students, feedback possibilities and interaction with the lecturers were provided. A weekly reflective video consultation session with the lecturers was also offered (university-internal *zoom platform*). This 45 to 60-minute live appointment served several didactical ideas:

1. The possibility of bridging the personal distance to the lecturers (*“personalisation principle”*) [12].
2. The opportunity of an optional learning- and time-structure as an aid to students who perceive a learning environment without distinct time and place commitments insufficient.
3. Consolidation of content (and not its repetition) in the sense of exam preparation.

**Figure 1:**
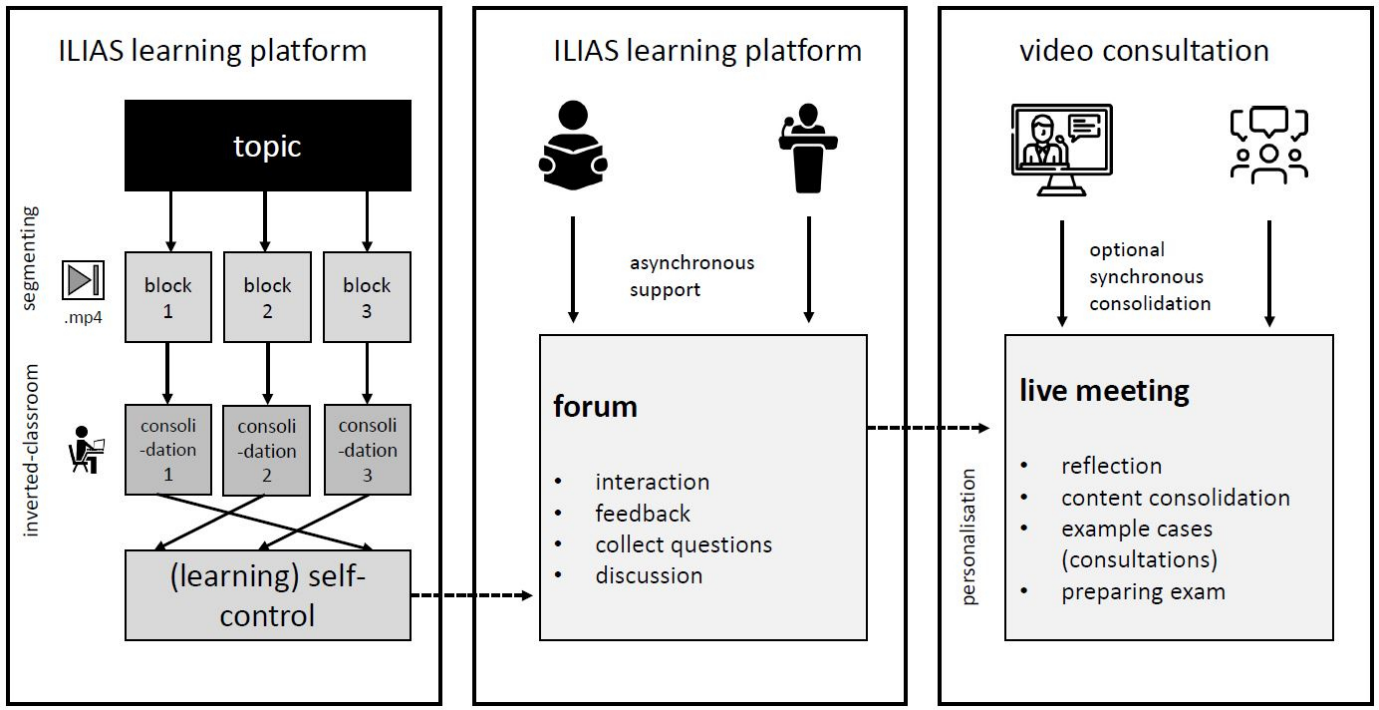
Overview of digital methods and content. The black arrows show the flow of digitally and asynchronously mediated content from the digital learning platform to the reflective video consultation. The dashed arrows show the transition where a change of digital didactics took place.

Thus, a digital variant of an inverted classroom concept was created [13]. This variant now offered the possibility of reflective learning in an asynchronous, location- and time-independent environment, a highly individual learning pace with constantly recurring points of contact with the lecturers via a supervised forum and virtual face-to-face consultation hours. Figure 1 (Fig. 1) shows the didactic teaching concept.

#### 2.2. Evaluation

After completing the lecture, an anonymous evaluation was carried out by the students via the learning platform. It contained self-developed items that were adapted to the digital concept of the lecture beyond the cumulative evaluation of the faculty. The overall impression of the lecture, didactic elements and methods, clarity of digital instructions as well as the desired future form of attendance were evaluated. The response format followed the German school grade principle (grades 1-6 (1=*“very good”*, 6=*“insufficient”*) or a 5-point Likert scale (1=*“fully agree”* to 5=*“fully disagree”*). Lastly, free text answers were also possible on further desired digital elements and criticism. The open, qualitative free-text evaluation was comprised of two questions:

1. What further digital elements would you like to see in the lecture for the future?
2. What other wishes (or criticism) do you have (feedback, further suggestions for improvement)?

Quantitative evaluation was carried out by exporting the data from the learning platform into an excel format for data preparation and depiction (Excel 2019, Microsoft Systems, Redmond, WA, USA). The free text answers were processed by qualitative content synthesis [14] through inductive category formation with the help of the software QCAmap [https://www.qcamap.org/].

## 3. Results

### 3.1. Quantitative evaluation

**Figure 2:**
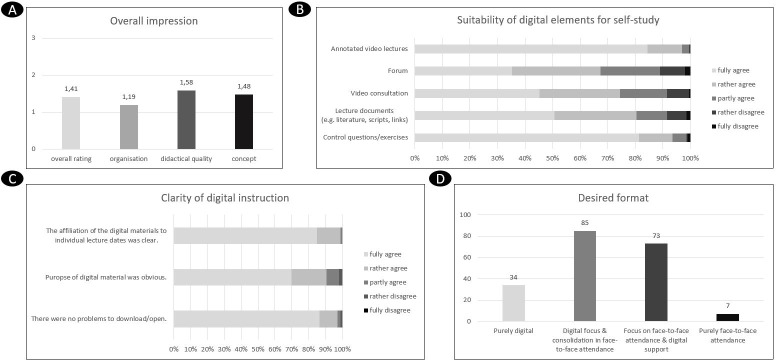
Overview of quantitative results a) Overall evaluation of the lecture “General Practice” within two digital semester cohorts; “How do you evaluate [overall] the digital lecture general practice?”; German school grade system, N=199 b) Suitability of digital elements for self-study; “I consider the following digital element to be suitable for supporting my self-study:”; 5-point Likert scale, N=199 c) Clarity of digital instruction; “Please indicate to what extent you agree with the following statements.”; 5-point Likert scale, N=199 d) Desired format; “Which teaching method do you feel is appropriate for the general practice lecture?”; option selection; N=199

The students rated the overall impression of the lecture according to German school grades (response rate *N=199/343; 58.0%*, see figure 2 (Fig. 2)) as “very good” on average (*M=1.41, SD=.57*). Digital elements and methods were perceived as predominantly suitable for supporting self-study. The digital lecture videos (*M=1.19, SD=.49*) were rated best in comparison to the forum (*M=2.11, SD=1.05*), which offers potential for improvement. The instructions for digital use were rated as mostly clear (*M=1.42, SD=.72*) and unproblematic (*M=1.18, SD=.50*). The predominantly desired future teaching and learning formats for the general practice lecture were *“Digital focus & consolidation in face-to-face attendance”* (42.7%; N=85) and *“Focus on face-to-face attendance & digital support”* (36.7%; N=73). Only 3.5% of the students wanted a pure face-to-face attendance.

#### 3.2. Qualitative evaluation

**Figure 3:**
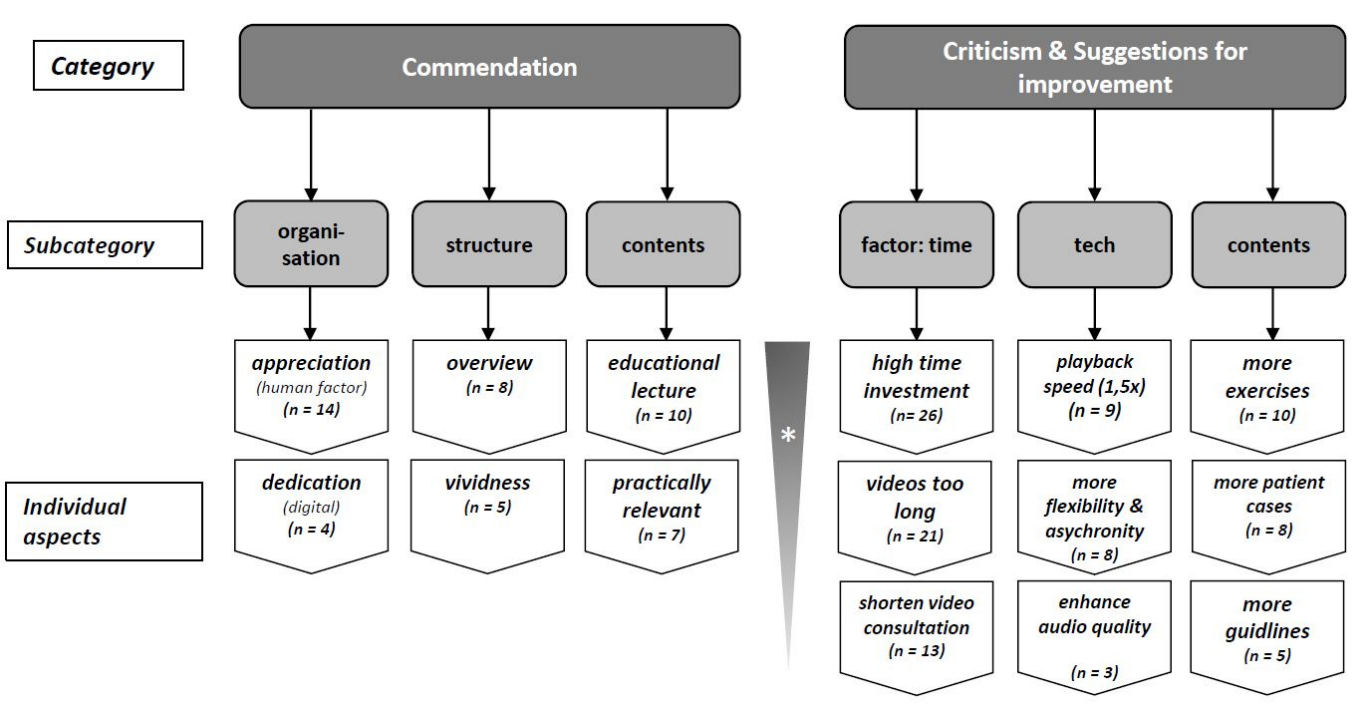
Overview of qualitative results after inductive content synthesis *Weighting of individual aspects descending from base to peak according to number of mentions (n)

Figure 3 (Fig. 3) shows the categories, subcategories and individual aspects of the free-text comments of the two open questions prepared according to inductive content synthesis. Two main categories “commendation” and “criticism and suggestions for improvement” were created. For each main category, three subcategories were formed, each comprising up to three individual aspects. The individual aspects were weighted according to number of mentions in a descending order.

## 4. Discussion

The quantitative evaluation of two semester cohorts in general practice shows a very good acceptance of asynchronous, digital teaching by the students during the pandemic. A demand for blended learning teaching concepts was particularly prominent. There was little demand for digital-only or pure face-to-face events with physical attendance for future teaching in general practice. The qualitative statements support the quantitative evaluation, especially in the subcategories of organisation, structure and content. However, distinct potential for improvement of the teaching concept was identified. The most profound criticism by the students was, that time investment was perceived as too high. Additionally, times of distanced teaching and learning show a demand for even more content-related discussion and active practice with the presented content. Technical aspects such as the demand for an individual playback speed of video and audio material play a minor role.

### 4.1. Factor: time

Although asynchronous, digital teaching allows students a lot of flexibility, an individual learning pace and locational independence in clinical teaching, this concept offers potential for improvement. The students’ main criticism was the increased time factor for completing the lecture. Both the preparation and consolidation phases should be considered when calculating the time required to not create an additional time burden on students with a "digital on top" measure. Considering the time required for content preparation (45-60 min.), consolidation through the video consultation (45-60 min.) and self-study consolidation phase through exercises and control questions (5-15 min.), the workload appears to be significantly higher than two SWS. From a didactic point of view, however, not only the pure time spent during the semester should be weighed against each other, but also the amount of time effectively used by the students. This concept translocated the main part of in-depth self-study and exam preparation to a phase together with the lecturers and digital connection and guidance. Following this, self-study can be flexibly structured, and preparation phases for the exam can also be effectively disburdened for the students. Particularly passive content transfer holds potential for demotivation with digitalisation [15] and must shift towards active handling of content, reflective consolidation and practical application. Faculty learning portals should also implement common individualisation functionalities (e.g. for individual playback speeds) in order to strengthen digital teaching technically as well.

#### 4.2. Practical teaching

A desire for more practice-oriented teaching is depicted by the demands for more guideline work, exercises and control questions together with the lecturers and practical examples. On the one hand, this illustrates the potential for teaching general practice content based on distinct situations in the actual field of work. On the other hand, the desire for active and reflective handling of information shows that too much time is still given to theoretical information transfer. This is particularly relevant because reflective consolidation offers potential during the semester and can thus significantly relieve the exam preparation time in self-study. The presented concept offers adjustment potential for a more effective use of students’ learning time.

#### 4.3. Bypass physical distance

The concept offers the opportunity to motivate students and grant flexibility in the digital medical curriculum [16]. Physical distance can be proactively addressed through a digital consultation hour or an asynchronous forum, thus favouring motivating self-learning strategies. The evaluated appreciation (in terms of the human and digital engagement during the pandemic) shows that an emotional connection of students to an asynchronously and digitally taught clinical subject can be generated [17]. Digital teaching can also be successfully used in courses with patient contact [18], [19].

#### 4.4. Accelerate self-study

Students rate the various digital elements differentiated in their suitability for self-study. The content-conveying annotated videos and the control questions are perceived as most suitable for self-study. More in-depth and reflective elements such as further reading and video tutorials score a little lower in this respect. The interactive forum is still perceived as suitable by more than 70% of the students, but drops in its suitability for self-study as a digital method. Important implications for digital teaching according to the inverted classroom concept within medicine arise:

1. Medical students should be confronted with the inverted classroom concept at an early stage, as the focus of students, especially in the first clinical semester, remains on purely content-based learning objectives in clinical subjects. The shift to suitable self-learning strategies should occur early, as these strategies remain an important basis for subsequent continuing medical education.
2. Digital elements must be evaluated with regard to their suitability for self-study. Digital elements in particular, such as consultation hours and the forum, must offer clear and tangible additional value for students in terms of consolidation and exam preparation. Otherwise they will be perceived as mere digital delivery of additional content. Open communication of expectations, teaching concept and didactic methods can help students to be more satisfied with digital teaching [20].

#### 4.5. Quo vadis – blended learning in medicine?

The new normality [21] requires a review of all preclinical and clinical subjects with regard to their potential for digitalisation. As our preliminary investigation, the present paper and another study show, the focus of digital teaching concepts in the future will be particularly on blended learning teaching concepts [6], [17]. However, lecturers were initially confronted with an increased time requirement to develop digital concepts, which resulted in additional burdens [22], [23]. The recognition of digital teaching (e.g. teaching effort calculation, digital didactics training) by the faculties is an important step towards recognising and promoting digital teaching achievements. This is also necessary so that the increased workload for students and lecturers in pilot semesters does not act as a deterrent leading to a post-pandemic return to the familiar, purely content-based formats in medicine. The present work shows that students were very satisfied with a structured and openly communicated asynchronous digital teaching concept in the inverted classroom model in general practice. For the future, students would like to see blended learning concepts. The digital flexibility should be maintained, and the phases of content transfer should be shortened in favour of practical, in-depth work together with the lecturers.

## 5. Limitations

This paper reports an evaluation of two semester cohorts in general practice at a Bavarian university in medicine. The study can therefore initially provide a report of the status quo for the subject of general practice. A valid comparison of the concept within the faculty and with other universities is not feasible due to the heterogeneity of teaching concepts. Moreover, in the case of voluntary evaluation, sample bias due to varying willingness to participate cannot be excluded. 

## Notes

### First authorship/author contributions

The authors Piet van der Keylen and Nikoletta Zeschick contributed equally to the present work and share the first authorship.

PK and NZ were responsible for manuscript preparation, collection and analysis of data and development of the evaluation. AL was responsible for the extraction, processing and presentation of the data. TK and MR revised and edited the manuscript. MR and NZ developed the evaluation. PK, MR and TK were mutually responsible for the development and implementation of the teaching idea. The present thesis was written in (partial) fulfilment of the requirements for obtaining the doctoral degree Dr. rer. biol. hum. for NZ. 

#### Data availability and data protection

All primary data collected can be requested in anonymised form from the authors. The anonymous collection of data with an internal, password- and access-protected learning platform ensured at all times that only participants of the specific event were able to evaluate. At no time was it technically possible to trace the data back to individual participants.

## Acknowledgements

We would like to sincerely thank the students of the "covid cohorts" being available for the evaluation and thus the completion of the present work. The authors would like to thank the medical faculty for its advice on digital implementation in the context of the pandemic conditions.

## Competing interests

The authors declare that they have no competing interests.
